# Quality assessment of buccal versus blood genomic DNA using the Affymetrix 500 K GeneChip

**DOI:** 10.1186/1471-2156-8-79

**Published:** 2007-11-08

**Authors:** Jessica G Woo, Guangyun Sun, Mary Haverbusch, Subbarao Indugula, Lisa J Martin, Joseph P Broderick, Ranjan Deka, Daniel Woo

**Affiliations:** 1 University of Cincinnati College of Medicine, 321 Albert Sabin Way, Cincinnati, OH 45267, USA; 2 Cincinnati Children's Hospital Medical Center, 3333 Burnet Avenue, Cincinnati, OH 45229, USA

## Abstract

**Background:**

With the advent of genome-wide genotyping, the utility of stored buccal brushes for DNA extraction and genotyping has been questioned. We sought to describe the genomic DNA yield and concordance between stored buccal brushes and blood samples from the same individuals in the context of Affymetrix 500 K Human GeneChip genotyping.

**Results:**

Buccal cytobrushes stored for ~7 years at -80°C prior to extraction yielded sufficient double stranded DNA (dsDNA) to be successfully genotyped on the Affymetrix ~262 K *NspI *chip, with yields between 536 and 1047 ng dsDNA. Using the BRLMM algorithm, genotyping call rates for blood samples averaged 98.4%, and for buccal samples averaged 97.8%. Matched blood samples exhibited 99.2% concordance, while matched blood and buccal samples exhibited 98.8% concordance.

**Conclusion:**

Buccal cytobrushes stored long-term result in sufficient dsDNA concentrations to achieve high genotyping call rates and concordance with stored blood samples in the context of Affymetrix 500 K SNP genotyping. Thus, given high-quality collection and storage protocols, it is possible to use stored buccal cytobrush samples for genome-wide association studies.

## Background

While blood is considered the optimal source for DNA, inclusion of a blood draw may deter study participation [1]. Buccal cytobrush collection is a simple, painless procedure that allows for effective DNA sampling from a large population, and has been used in several large epidemiologic studies [2,3]. However, concerns regarding the use of buccal brushes have included the lower quantity of genomic DNA isolated [4], lower quality of DNA [4,5], and the fidelity of results from buccal brushes compared with blood samples [5-7]. In addition, there is a concern that older buccal brush samples may not yield as high-quality results as fresh samples [8].

The advent of large scale genotyping platforms has also resulted in a reduction in the amount of DNA required. The Affymetrix 500 K GeneChip requires only 250 ng of total genomic DNA per chip, 500 ng total, and this DNA quantity has not changed with the recent release of the Affymetrix 5.0 and 6.0 chips, which enable genotyping up to 1.8 million genetic markers [9-11]. Thus, the DNA requirements of the Affymetrix chips are well below the expected yield of total DNA for buccal samples. As the Affymetrix system uses restriction enzymes to recognize a particular double-stranded DNA (dsDNA) sequence, we elected to examine the dsDNA yield from buccal cytobrushes, rather than total DNA including both single and double stranded DNA.

We designed experiments to determine the genotyping performance of the Affymetrix 500 K GeneChip on stored blood and buccal brush samples from the same patient. In particular, we tested whether buccal brushes stored for over 5 years would yield sufficient quantities of genomic dsDNA for genome-wide genotyping, and whether buccal brush genomic DNA would result in comparable genotype call rates and concordance with matched blood DNA.

## Results

### dsDNA Yield and Call Rates

All eight buccal samples yielded sufficient dsDNA to be typed by the *NspI *Affymetrix 250 K chip, ranging from 536 to 1047 ng dsDNA (Table 1). Genotyping call rates for blood samples averaged 98.4%, and for buccal samples averaged 97.8% (Table 1), a 0.6% difference in call rates.

**Table 1:** dsDNA Yield and BRLMM Call Rates by Sample Type

	Technical Replicates – Blood			Biologic Replicates – Buccal Brushes			
		Replicate 1	Replicate 2	Replicate 1		Replicate 2	

Individual	dsDNA (ng)*	Call Rate (%)	Call Rate (%)	dsDNA (ng)	Call Rate (%)	dsDNA (ng)	Call Rate (%)

A	44000	97.5	99.3	831	96.8	765	97.2
B	25410	98.8	98.6	758	98.5	846	98.9
C	39440	98.9	97.0	536	99.2	612	99.6
D	29520	98.0	99.3	768	96.8	1047	95.3

* Yield is reported for blood prior to aliquotting, not for each aliquot

### Concordance between Matched Blood and Buccal Samples

Comparing blood samples (technical replicates), the average concordance was 99.2% (over an average of 254,729 SNPs), which represents our 'gold standard' (Table 2). Considering all four possible buccal-blood comparisons, concordance averaged 98.8% (over an average of 253,028 SNPs), a 0.4% difference in concordance. For simplicity, Table 2 presents only two of the four buccal-blood comparisons per individual. Kappa statistics between blood and buccal calls ranged from 0.96 to 0.99, which represent near perfect agreement. Concordance between paired buccal samples averaged 99.0% over 251,772 SNPs. There was no difference in these results by replicate.

**Table 2:** Kappa Statistics and % Concordance of Called Genotypes

	Blood-Blood			Blood-Buccal (Replicate 1)			Blood-Buccal (Replicate 2)		
Individual	N (%) SNPs *	Kappa	% Conc.	N (%) SNPs *	Kappa	% Conc.	N (%) SNPs *	Kappa	% Conc.

A	254,510 (97.0)	0.98	99.0	248,352 (94.7)	0.97	98.2	253,642 (96.7)	0.98	98.7
B	256,346 (97.7)	0.99	99.6	256,100 (97.6)	0.99	99.6	256,431 (97.7)	0.99	99.5
C	252,243 (96.2)	0.98	98.8	257,629 (98.2)	0.99	99.6	253,471 (96.6)	0.97	98.3
D	255,817 (97.5)	0.99	99.4	250,054 (95.3)	0.98	98.7	248,511 (94.7)	0.96	97.3

Kappa statistics and % concordance calculated from genotypes called in both samples

* N (% of total) SNPs with genotype calls in both samples; Total SNPs on *NspI *chip = 262,314

## Discussion

This report demonstrates the utility of buccal brush genomic dsDNA in genome-wide SNP genotyping using the Affymetrix platform. We found buccal cytobrush genomic dsDNA is available in sufficient quantity and quality to be used for genome-wide (~262 K) SNP genotyping. While dsDNA yield and BRLMM call rates are often lower for buccal brushes than blood samples, the buccal brushes nonetheless exceeded 95% call rates in all samples.

The yield of genomic buccal DNA we obtained, ranging from 536 to 1047 ng of double-stranded DNA, is difficult to compare to previous studies. Previous studies have reported a large range of yields from cytobrushes, from ~0.5 to 12.66 μg total DNA [4,5,12,13], and none report dsDNA yields. However, our dsDNA yields were generally lower than these reported total DNA yields, likely due to the proportion of dsDNA versus total. However, the high genotyping call rates in the current study suggest the yield of dsDNA from buccal samples generally exceeds an undefined minimum required for high-quality results.

We also report that concordance rates for blood and buccal brush samples from the same individual approach those obtained by matched blood samples, suggesting excellent fidelity of buccal brush genotyping in this context. This is particularly notable, as the blood samples represent technical replicates, with a single collection and extraction, while the buccal samples are biological replicates, with two separate collection and extraction processes. Thus, the buccal samples include variability introduced by differences in collection and DNA extraction that does not exist in our blood samples. Previous studies of a smaller number of SNPs (~1 K to 56 K) on the Illumina and Affymetrix platforms also noted high concordance rates between genomic buccal and blood DNA [14,15]. A recent study comparing whole genome amplified buccal samples with blood genotyping on the genome-wide 300 K Illumina platform also found very high concordance [16].

It should be noted that the BRLMM calling algorithm typically requires image files from 100 unique individuals to create an informative clustering algorithm [17]. However, in the current study, we analyzed only 16 image files from 4 unique individuals (e.g., 2 buccal samples and 2 blood aliquots from each individual), and only ~11% of SNPs had an informative prior for clustering. Thus, it is likely that genotype call rates and concordance would improve with a larger sample size. In addition, it should be noted that the technical replicates (blood samples) had less than perfect call rates and concordance. Thus, given this baseline error rate, the buccal brush genotype call rates and concordance are comparable to blood.

Strengths of this study include the collection of buccal cytobrushes by trained research nurses and uniform long-term storage procedures. Buccal brushes are sometimes difficult to use and may result in low DNA yields, especially when individuals self-collect the sample [12]. Improper collection may result in low DNA yields and bacterial contamination, and poor storage protocols may result in the degradation of the collected sample. While we did not specifically quantitate non-human DNA in our samples, our quantitation was restricted to dsDNA, eliminating the possibility of bacterial (single-strand) DNA being included in our yields. Also, the high call rates we and others have reported for buccal samples suggest that bacterial contamination may, in fact, be a minor issue, perhaps due to the human-specific probes employed in large-scale genotyping. The applicability of using buccal brush samples in genome-wide association studies may therefore depend heavily on the initial quality of the buccal brush collection, although this question was beyond the scope of the current study.

## Conclusion

The current study suggests several methodologic recommendations for using buccal cytobrushes for genome-wide association studies or similar large-scale SNP genotyping. First, buccal brushes should be collected using high-quality procedures by trained study personnel, if possible. All buccal brushes in the current study were collected using such standardized methods, resulting in excellent performance. Second, because some buccal samples had low yields, it is recommended that several buccal brushes be collected on each patient. In the current study, we ultimately obtained high-quality genotyping for each patient, but one individual would have required three to four brushes to be extracted to achieve those results.

Finally, quantitation of dsDNA is important, especially for the Affymetrix GeneChip, which relies on restriction enzymes. Buccal samples with dsDNA concentrations <50 ng/μl had low call rates, suggesting caution in genotyping samples below the 50 ng/μl DNA threshold recommended by Affymetrix. Future research will be necessary to determine the minimal dsDNA required for genome-wide genotyping in this context.

In conclusion, buccal cytobrushes stored long-term result in sufficient dsDNA to achieve high genotyping call rates and concordance with stored blood samples in the context of Affymetrix 500 K SNP genotyping. Thus, given high-quality collection and storage protocols, it is possible to use stored buccal brush samples for genome-wide association studies.

## Methods

### Blood and Buccal Swab Collection

The Genetic and Environmental Risk Factors for Hemorrhagic Stroke (GERFHS) study is a large case-control study of hemorrhagic stroke in the Cincinnati, Ohio region. This study protocol was approved by the Institutional Review Board of the University of Cincinnati, and all subjects provided written informed consent for genetic testing. To maximize participation and thereby maximize representativeness of the cohort, buccal cytobrush collection was performed on the majority of subjects. Buccal brushes for genetic analysis were collected beginning in 1997, and in mid-2000, the option of collection of blood samples was added to the protocol. Four individuals enrolled in 2000 or 2001 had both buccal brushes and blood samples collected, and were selected for this analysis. Four buccal brushes were collected on each participant (two right cheek, two left cheek) using CYTO-PAK Cytosoft Brushes (Medical Packaging Corp., Camarillo, CA), and two of the four collected buccal brushes were used for this analysis, except as noted below.

Study nurses were trained to collect buccal cytobrush samples by the following procedure: The subject would rinse their mouth with water gently prior to brush collection. Each brush was inserted into the subject's mouth and twirled firmly for 30 seconds against the subject's cheek in an up and down motion. The nurse returned the brush into the plastic tube and sealed the tube with the subject ID label. After all four brushes were collected, the sealed brushes were sealed again in an envelope labelled with the ID number and date and time of collection. These envelopes were stored in sealed freezer bags and stored at -80°C until they were transferred to the lab for DNA extraction. Blood samples were drawn by hospital nursing personnel using two 10 ml purple top tubes with EDTA solution from subjects during their hospital stay. The blood samples were spun down, DNA extracted and the DNA was stored at -80°C in the lab for future analysis.

### DNA Extraction and Genotyping

For each buccal cytobrush, DNA was extracted using PureGene kits (Gentra Systems, Inc. Minneapolis, MN), resulting in eight separate buccal brush DNA samples for analysis (biological replicates). DNA was extracted from a single blood sample for each individual by Molecular Diagnostics Laboratories, Inc., and then separated into two aliquots for analysis (technical replicates). Throughout the paper, duplicate blood or buccal samples are designated either "replicate 1" or "replicate 2" to distinguish them; however, all samples for each individual were genotyped at the same time.

To extract blood DNA, a standard Proteinase K procedure was used. Briefly, this procedure included isolation and lysing white blood cells, treatment with Proteinase K (20 mg/μl), precipitation and washing DNA with isopropanol and resuspension of DNA in 500 μl of TE buffer. The Quant-it dsDNA HS assay kit and Qubit fluorometer (Invitrogen; Carlsbad, CA) were used for quantitating double-stranded DNA (dsDNA) in solution. This is a highly sensitive, dsDNA-specific assay employing a fluorescent nucleic acid stain.

Five buccal brush samples had initial concentrations of dsDNA <40 ng/μl (range: 3.4 – 39.8 ng/μl), and additional DNA was extracted from the tube that originally contained the buccal cytobrush. After this procedure, two buccal brushes (both from individual C) still had dsDNA concentrations <50 ng/μl (replicate 1: 40.4 ng/μl, replicate 2: 30.8 ng/μl). These samples were initially analyzed using 202 ng and 154 ng of dsDNA per GeneChip, respectively, but call rates were quite low (95% and 89%, respectively). The two remaining buccal brushes for individual C were then extracted and genotyped, and results from these latter two buccal brushes are reported.

Genotyping was performed on the Affymetrix GeneChip 3000 platform. We used the *Nsp1 *chip, which is an array of ~262 K SNPs. The recommended protocol as described in the Affymetrix manual was followed. All DNA samples were normalized to 50 ng/μl. Then, 5 μl (250 ng) of dsDNA was digested with *NspI *and ligated to adapters using T4 DNA ligase. Samples were then PCR amplified using TITANIUM Taq polymerase on an ABI9700 machine. PCR products were purified using the Clontech purification kit followed by fragmentation. Samples were then injected into cartridges, hybridized, washed and stained.

Mapping array images were obtained using the GeneChip Scanner 3000. Genotypes were called using the BRLMM software, analyzing all .cel files together. Any sample with a genotype call rate <95% was considered a QC failure, which would result in the sample being re-extracted and/or re-genotyped in a full-scale genetic study.

### Statistical Methods

For each individual, two buccal samples (biological replicates) and two blood aliquots (technical replicates) were run, such that matched blood and buccal samples as well as matched blood samples from the same individual could be compared. Four possible comparisons of blood and buccal samples were analyzed: 1) blood replicate 1 with buccal replicate 1; 2) blood replicate 1 with buccal replicate 2; 3) blood replicate 2 with buccal replicate 1; and 4) blood replicate 2 with buccal replicate 2. Concordance of genotype calls between samples from the same individual was evaluated using % concordance and the Kappa statistic, which measures the agreement between methods exceeding that expected by chance. Percent concordance and Kappa statistics were calculated only among genotypes called in both samples being compared, excluding missing data. P ≤ 0.05 was considered significant.

## Authors' contributions

JGW conducted statistical analysis and drafted the manuscript. GS conducted the DNA extraction and genotyping and assisted with the drafting of the manuscript. MH collected the samples and assisted with manuscript development. SI participated in sample processing and genotyping. LJM assisted with statistical analysis and manuscript development. JPB, RD and DW participated in the study design and manuscript development. All authors read and approved the final manuscript.
